# Supplementary figures and images for: Citizen science reveals unexpected solute patterns in semiarid river networks
Source: PLoS One. 2021 Aug 19;16(8):e0255411. doi: 10.1371/journal.pone.0255411 (PMC8376020; doi:10.1371/journal.pone.0255411)

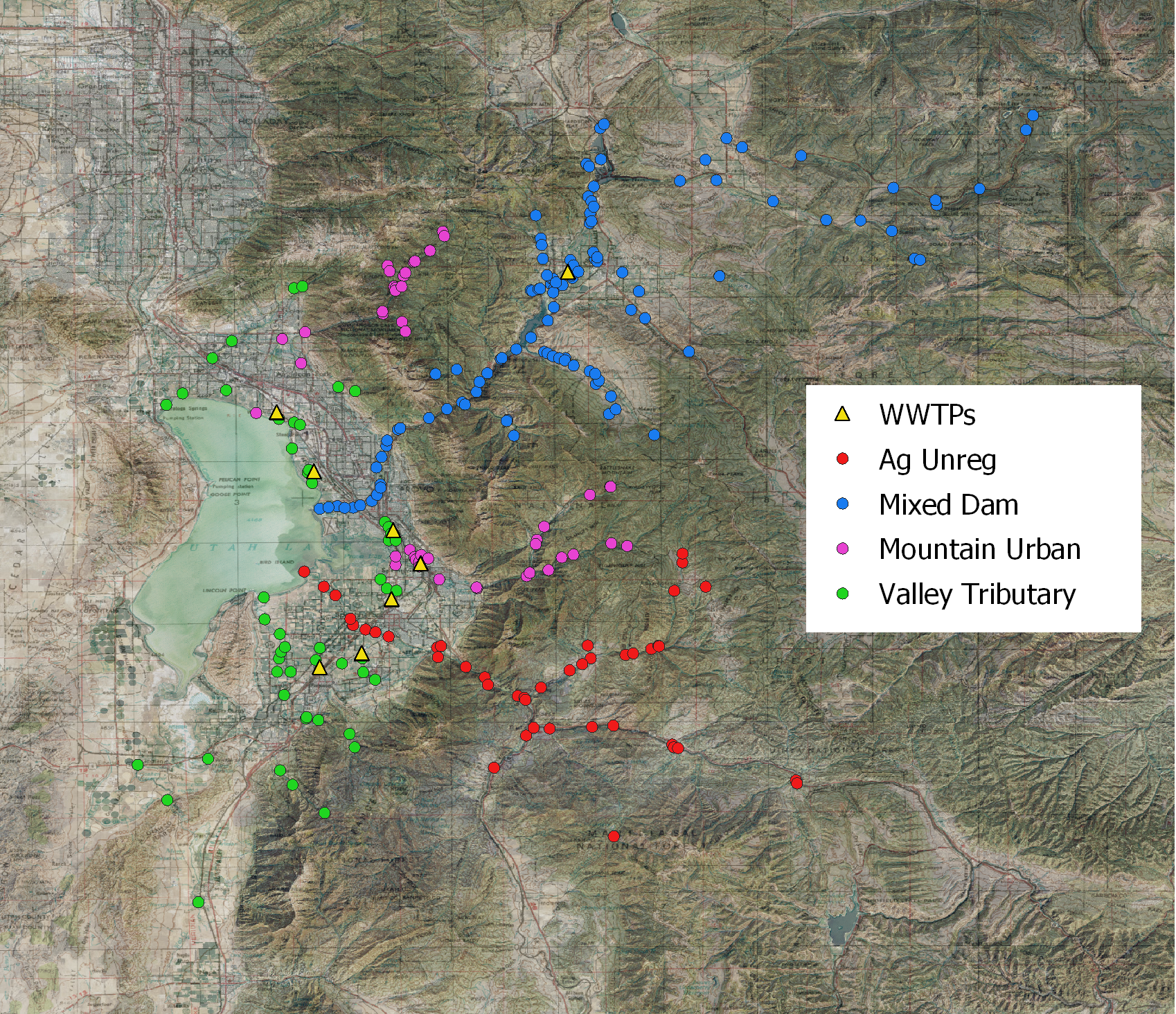

Supplement: S1 Fig — Points colored by land use and hydrologic modification category (red = Agricultural unregulated, green = Valley tributaries, blue = Mixed dammed, purple = Mountain urban). Yellow triangles represent wastewater treatment plants. Basemap source: USGS National Map and OpenStreetMap. (TIF) [file pone.0255411.s001.tif]
