# Supplementary material for: Citizen science reveals unexpected solute patterns in semiarid river networks
Source: PLoS One. 2021 Aug 19;16(8):e0255411. doi: 10.1371/journal.pone.0255411 (PMC8376020; doi:10.1371/journal.pone.0255411)
Supplement: S2 Fig — Includes sampling instructions and datasheet for recording sample information. Instruction sheet shows photo of co-author GML demonstrating sampling technique. (DOCX) [file pone.0255411.s002.docx]

1. Safety (literally) first! No sample is worth risking your life. Drive safely and be aware around the water.
2. Go to your site (**Pro tip:** you can enter the GPS coordinates into Google Maps on your phone). If it is not possible to safely and legally access the stream or lake at the exact location indicated on the map, find a nearby spot and make a note.
3. Before getting your hands wet, write the site number and the time on the bottles and fill out the data sheet.
4.
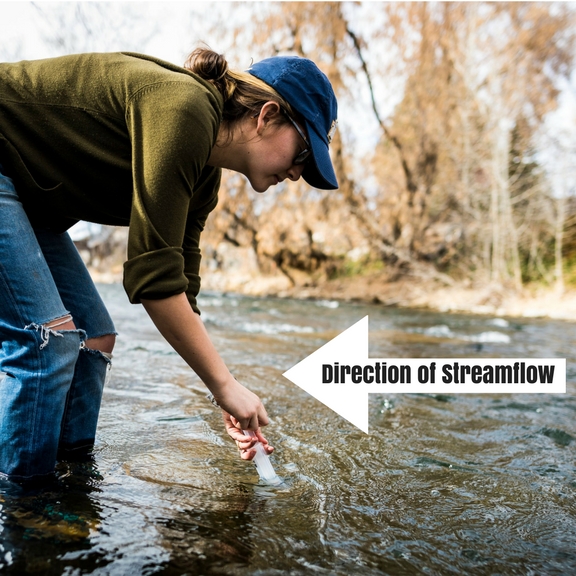
Collect your water samples! Find a safe place along the bank or in the channel and face upstream (see picture to the right)
   1. For the **little brown bottle**
      1. Rinse the syringe three times
         1. Put the tip of the syringe a few inches below the water’s surface
         2. Suck up water to fill the syringe
         3. Squirt the water out downstream or towards shore
      2. Fill the rinsed syringe completely and filter the water sample
         1. Push your filter firmly onto the tip of the full syringe and push 10 mL of water through the filter onto the ground to rinse the filter
         2. Push the remaining water through the filter into the little brown bottle
   2. For the **big clear bottle**
      1. Scoop up unfiltered water from a few inches below the surface
   3. Make sure the bottles are capped tightly, put them back in the bag, and put the bag in the cooler.
   4. Do a victory dance
5. Take a moment to enjoy nature then on to the next site!
6. Return samples, materials, and completed data sheets to the drop off spot by 4pm


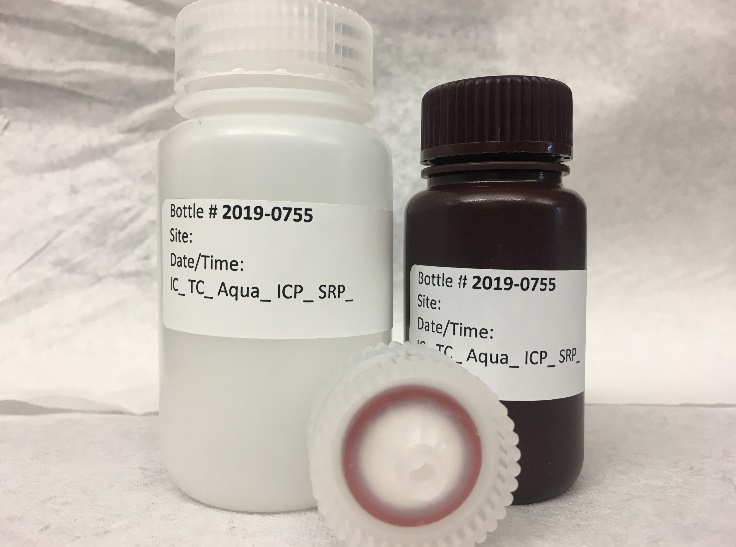


**What do we do with your sample?**

Thanks to you and other citizen scientists collecting samples today, we can capture a high-resolution “snapshot” of the water chemistry in all the tributaries to Utah Lake. We will use this dataset to identify sources of pollutants and assess how human restoration and natural ecosystem processes like plant uptake and denitrification can help clean up the water. Specifically we will perform the following analyses: water isotopes, carbon, nitrogen, phosphorus, ions, and organic matter properties. Check the website to access the data yourself: **tiny.cc/f2ah**

# **How do we use our water?**

Water resources are fluctuating due to climate change, population growth, nutrient loading into water systems, debates over water usage rights and many more factors. While this can be worrying, we can and need to use what resources we have more effectively. This chart shows current water usage in Utah:


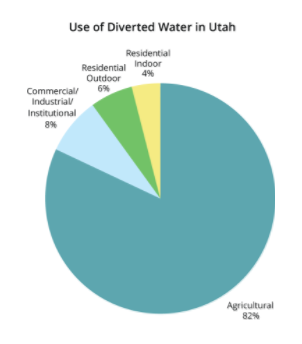


Figure 1 http://www.utahfoundation.org/reports/flowing-toward-2050-utahs-water-outlook/

**Five Things You Can Do:**

1. Residential outdoor water consumption accounts for 6% of our water usage. Mow your lawn to be 1.5-3” and leave the clippings as a fertilizer.

2. Agriculture is the largest water user of all categories, so this is where the biggest impact can be made. Eat lower on the food chain; try having more plants and less meat and dairy, and buy locally when possible.

3. Take shorter showers, and minimize running the dishwasher, washing machine, faucets, showerheads, and other water sources when they’re not being used.

4. Minimize driving by turning to other transportation sources (walking, biking, public transportations, carpooling, etc.)

5. Give recycling or composting a try!

**Name of citizen scientist:**

**Email:**

| **Bottle** | 2018- |
| --- | --- |
| **Site number (three digits)** |  |
| **Time sampled** |  |
| **Notes on weather conditions** |  |
| **Notes on access to site (circle one: 1= very easy, 5 = very challenging)** | **1 2 3 4 5** (if 5, please describe) |
| **Other notes** (any obvious sources of pollution, garbage, construction, etc.) |  |

| **Bottle** | 2018- |
| --- | --- |
| **Site number (three digits)** |  |
| **Time sampled** |  |
| **Notes on weather conditions** |  |
| **Notes on access to site (circle one: 1= very easy, 5 = very challenging)** | **1 2 3 4 5** (if 5, please describe) |
| **Other notes** (any obvious sources of pollution, garbage, construction, etc.) |  |
