# Supplementary material for: Citizen science reveals unexpected solute patterns in semiarid river networks
Source: PLoS One. 2021 Aug 19;16(8):e0255411. doi: 10.1371/journal.pone.0255411 (PMC8376020; doi:10.1371/journal.pone.0255411)
Supplement: S1 Table — (DOCX) [file pone.0255411.s003.docx]

**S1 Table. ANOVA test comparing solute concentrations in streams from different land use categories over three synoptic sampling events in the Utah Lake watershed.** Asterisks denote significant *p*-values: '***' < 0.001; '**' <0.01; '*' < 0.05; '.' < 0.1 .

Df Sum Sq Mean Sq F value Pr(>F)

Season 2 10.2 5.09 5.896 0.00279 **

Solute 5 0.1 0.02 0.023 0.99978

Category 3 248.8 82.94 96.055 < 2e-16 ***

Season:Solute 10 69.6 6.96 8.057 6.37e-13 ***

Season:Category 6 10.6 1.76 2.042 0.05699 .

Solute:Category 15 56.3 3.76 4.349 4.03e-08 ***

Season:variable:Category 30 27.0 0.90 1.043 0.40212
