# Supplementary material for: Citizen science reveals unexpected solute patterns in semiarid river networks
Source: PLoS One. 2021 Aug 19;16(8):e0255411. doi: 10.1371/journal.pone.0255411 (PMC8376020; doi:10.1371/journal.pone.0255411)
Supplement: S2 Table — (DOCX) [file pone.0255411.s004.docx]

**S2 Table. Distribution of leverage values in each subwatershed for each solute.**

|  | **DOC** | **PO_4_^3-^** | **TN** | **DIN** | **Cl^-^** | **SO_4_^2-^** |
| --- | --- | --- | --- | --- | --- | --- |
| **Percent of subwatersheds which were:** |  |  |  |  |  |  |
| **Sinks or neutral** | 44 | 39 | 48 | 44 | 72 | 65 |
| **Sources** | 56 | 61 | 52 | 56 | 28 | 35 |
| **Highly influential (>100% leverage)** | 10 | 12 | 12 | 10 | 1 | 2 |
| **Low influence (<25% of catchment concentration)** | 69 | 59 | 68 | 69 | 85 | 84 |
