# Supplementary material for: Citizen science reveals unexpected solute patterns in semiarid river networks
Source: PLoS One. 2021 Aug 19;16(8):e0255411. doi: 10.1371/journal.pone.0255411 (PMC8376020; doi:10.1371/journal.pone.0255411)
Supplement: S3 Table — Asterisks denote significant p-values: ’***’ < 0.001; ’**’ <0.01; ’*’ < 0.05; ’.’ < 0.1. (DOCX) [file pone.0255411.s005.docx]

**S3 Table. ANOVA test comparing spatial persistence values for solutes measured in different land use categories over three synoptic sampling events in the Utah Lake watershed.** Asterisks denote significant *p*-values: '***' < 0.001; '**' <0.01; '*' < 0.05; '.' < 0.1 .

Df Sum Sq Mean Sq F value Pr(>F)

Solute 10 5.245 0.5245 21.542 < 2e-16 ***

Category 3 1.750 0.5834 23.960 1.74e-11 ***

Season 3 0.365 0.1216 4.993 0.0030 **

Solute:Category 30 2.567 0.0856 3.514 2.21e-06 ***

Category:Season 9 1.137 0.1264 5.190 1.14e-05 ***

Solute:Season 30 1.278 0.0426 1.750 0.0229 *

Residuals 90 2.191 0.0243
